# Occurrence of postdural puncture headache—A randomized controlled trial comparing 22G *Sprotte* and *Quincke*

**DOI:** 10.1002/brb3.1886

**Published:** 2020-10-12

**Authors:** Ane Skaare Sjulstad, Francis Odeh, Farid K. Baloch, Diana Hristova Berg, Kathrine Arntzen, Karl B. Alstadhaug

**Affiliations:** ^1^ Nordland Hospital Trust Bodø Norway; ^2^ Institute of Clinical Medicine The Arctic University of Norway Tromsø Norway

**Keywords:** 22‐gauge Quincke (traumatic needle), 22‐gauge Sprotte (atraumatic needle), cerebrospinal fluid, diagnostic lumbar puncture, postdural puncture headache

## Abstract

**Objective:**

To assess the incidence of postdural puncture headache (PDPH) using 22‐gauge atraumatic needle (*Sprotte*, 22GS) compared with 22‐gauge traumatic needle (*Quincke*, 22GQ).

**Background:**

Diagnostic lumbar puncture (dLP) is commonly complicated by PDPH. Despite evidence to support the use of 22GS, European neurologists seem to keep using 22GQ.

**Methods:**

This was a randomized, double‐blind study. Adults (age: 18–60 years) scheduled for dLP were included. dLP and CSF acquisition were performed in accordance with highly standardized procedures. Patients were followed up on days 2 and 7.

**Results:**

In total, 172 patients were randomized and lumbar punctured, and 21 were excluded due to wrong inclusion (*n* = 11), needle switch (*n* = 7), failed dLP (*n* = 1), withdrawal (*n* = 1), and missed follow‐up (*n* = 1). Among the remaining 151 patients (mean age: 40.7 ± 12.4 years), 77 had dLP using 22GQ and 74 using 22GS. Incidence of PDPH among patients punctured with 22GS (18%) was significantly lower (*p* = .004) than among patients punctured with 22GQ (39%). Relative risk was 0.45, 95% CI 0.26–0.80. Patients with PDPH had significantly lower weight (*p* = .035), and there was no significant difference related to age (*p* = .064), sex (*p* = .239), height (*p* = .857), premorbid episodic migraine (*p* = .829), opening pressure (*p* = .117), operators (*p* = .148), amount of CSF removed (*p* = .205), or number of attempts (*p* = .623).

**Conclusions:**

The use of 22GS halves the risk of PDPH compared with 22GQ. This study provides strong support to make a change in practice where traumatic needles are still in regular use.

## Graphical abstract

Diagnostic lumbar puncture is commonly complicated by postdural puncture headache (PDPH), and several studies comparing the incidence of PDPH have confirmed a clear benefit of the atraumatic spinal needle. Despite this, neurologists seem to keep using the traumatic needle. This study provides strong support to make a change in practice where traumatic needles are still in regular use.

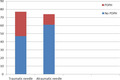

## 1 INTRODUCTION

Diagnostic lumbar puncture (dLP) is probably one of the most commonly performed invasive procedures in clinical medicine. It is easy to master, highly available, and permits direct access to the environment of the central nervous system (CNS). The procedure is essential, and the indications are many, to exclude subarachnoid hemorrhage in acute headache (Sjulstad & Alstadhaug, 2019), measure intracranial pressure, and investigate CNS infections and a large number of neurological disorders. dLP is generally safe, but a common complication is postdural puncture headache (PDPH), first described in 1898. (Bier, 1899) Several studies comparing incidence of PDPH when using atraumatic and traumatic needles (Arevalo‐Rodriguez et al., 2017; Nath et al., 2018) have confirmed a clear benefit of the atraumatic needle. Other benefits reported with using atraumatic needles are reduced need for epidural blood patch and treatment with intravenous fluids and analgesics. In other studies, reduction in the incidence of both mild and severe PDPH, any headache, nerve root irritation, and hearing disturbances has been shown. (Nath et al., 2018) It has earlier been pointed out that failure to switch from the traditional to the atraumatic needle was due to lack of communication (“diffusion of innovation”) rather than evidence and that neurologists compared with anesthetists are more prone to this. A common perception is that using atraumatic needles are more complex. Furthermore, the complexity of innovation affects the likelihood of its adoption. The first studies published showing a reduced incidence of PDPH following the use of atraumatic needles were published by anesthetists. Maybe that communication is different among the different specialities, with differences in medical practice and training. The anesthetists have a procedure‐based approach and are doing a lot of practical training, which may have facilitated the diffusion and adoption of atraumatic needles. (Davis et al., 2016).

In a Cochrane report from 2017, however, it was pointed out that the quality of evidence is moderate and that “further research is likely to have an important impact on our confidence in the estimate of effect.” In our opinion, poor evaluation of headaches, both prior to the dLP and afterward, is potentially one of the greatest biases in previous randomized controlled studies.

The 22G needle is considered most appropriate for dLP (Armon & Evans, 2005), and we wanted to do a new methodically robust study of the beneficial effect of atraumatic needle in dLP, hoping to convince the neurological societies to change practice where traumatic needles are still in regular use.

## 2 METHODS

### 2.1 Primary research question

Does the use of 22G atraumatic needle for dLP reduce the incidence of PDPH?

### 2.2 Standard protocol approvals, trial registration, and participant consents

The study was approved by the Regional Committee for Medical and Health Research Ethics (REK nord 2011/1083). The trial was registered in "ClinicalTrials.gov" in 2015, but the trial's progress and planning is publicly accessible back to 2011. (REK, 2011) An amendment for specific CSF analyses with re‐adjustment of sample size was approved in 2012, and there was a change of project leader in 2015. Except from that, the protocol has been unchanged. Written informed consent was obtained from all the patients before they were set up for a dLP.

Clinicaltrials.gov identifier: NCT02384031.

### 2.3 Patients

Eligible patients were scheduled to undergo a dLP as part of their clinical/diagnostic management, irrespective of this trial. All of them were recruited during their admission/visit to the Department of Neurology at Nordland Hospital in Bodø, Norway. They had to be between 18 and 60 years of age. The doctor performing the procedure informed the patients, and they got a consent form including study information.

Exclusion criteria were dementia, local skin infection over the proposed puncture site, and suspicion of raised ICP (papilloedema or results from cranial CT/MRI). Also thrombocytopenia, ongoing anticoagulant therapy, spinal column deformities, procedural complications, whereby needle type or size change was a requisite, and recent dLP (<7 days) led to exclusion. A headache history was obtained before inclusion, and subjects with chronic headache (≥15 days per month) or acute headache were also excluded.

### 2.4 Study design

**FIGURE 1:**
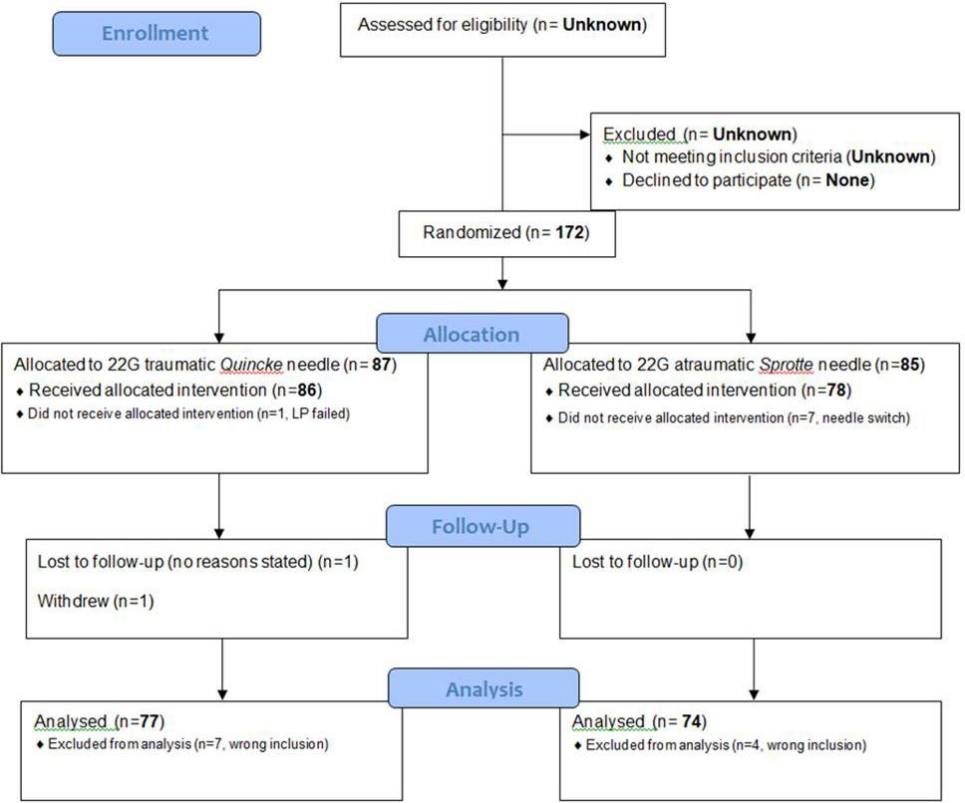
Flow diagram. Occurrence of postdural puncture headache—an RCT comparing 22G *Sprotte* and *Quincke*

Informed consenting patients were randomized in two groups based on needle type. The CONSORT flow diagram (Figure 1) shows the progress through this parallel‐designed trial where one group being lumbar punctured with Spinocan^®^ (Quincke) 22Gx3,5 needle, and the other group being lumbar punctured with Pencan^®^ (Sprotte), 22Gx3,5 needle. B. Braun Melsungen AG supplied the needles. The Unit for Applied Clinical Research at the Norwegian University of Science and Technology (NTNU) in Trondheim, an external and independent part of this project, ensured the randomization process and provided an Internet‐based application. Patients were randomized only once.

The procedure was performed in accordance with highly standardized and established procedures (see below). Patients were blinded to the randomization and needle type. The performing resident knew which needle was being used for obvious reasons. Physicians performing the dLP were not involved in patient follow‐up, which was performed by investigators blinded to the randomization. Patients were contacted by phone; if they still were admitted to the hospital, the interview was done bedside, on days 2 and 7 after the dLP. If the day occurred during a weekend or holiday, the interview was postponed to the nearest following weekday. A structured headache story was taken, and the patients were asked about the course if there had been interventions (emergency room contact, re‐admittance, bed rest, therapy, extended hospitalization, sick leave, and other complications). Patients who developed PDPH were treated in‐line with established and standardized protocols at the department. Given that PDPH has a natural history of spontaneous resolution within 1 week (Dripps & Vandam, 1954; Wadud et al., 2006), patients suffering from PDPH were treated conservatively for the first 7 days after the diagnosis. Based on symptom severity and patients’ responsiveness, it consisted of bed rest, caffeine, analgesics, and intravenous fluid. Subjects still suffering from severe and disabling headache, nonresponding of this treatment after 7 days, were treated with an epidural blood patch (EBP).

### 2.5 dLP procedure

The patient was positioned in a lateral decubitus position during the procedure. Some of the most anxious patients were treated with local anesthetics, lidocaine 1%. When using the traumatic needle, the bevel was parallel to the longitudinal axis of the spine during insertion. The needle was then slightly angled and aimed toward the navel. The quantity of CSF was determined by the analyses requested. The stylet was reinserted before the needle was withdrawn. If experiencing “dry tap,” the stylet was reinserted and the needle withdrawn, and another attempt was made, alternatively at another level. In some cases, the procedure was performed with the patient in a sitting position. Each patient had 30 min of bed rest after the procedure. Patients were included only on weekdays (Monday–Friday), between 07.30 a.m. and 4.00 p.m.

### 2.6 Outcomes

The primary outcome was the occurrence of PDPH. Secondary outcomes were potential risk factors for developing PDPH as listed in Table 1. Headaches, both prior to LP and on days 2 and 7 after the procedure, were diagnosed in accordance with the international criteria (Headache Classification Committee of the International Headache Society) (Headache Classification Subcommittee of the International Headache Society, 2004). Table 2 shows the criteria for PDPH.

**TABLE 1:** Demographic data and potential risk factors for developing postdural puncture headache

Included patients(*n* = 151)	PDPH (*n* = 43)	No PDPH (*n* = 108)	*p*‐value
Mean age, years ± *SD*	37.7 ± 12.0	41.9 ± 12.5	.064
Sex, males/females (%males)	15/28 (35)	49/59 (45)	.239
Weight, kg	72.5 ± 16.3	79.1 ± 17.6	.035
Height, m	1.73 ± 0.09	1.73 ± 0.09	.857
Mean BMI, kg/m^2^	24.1 ± 5.0	26.4 ± 5.3	.012
BMI obese (BMI ≥ 30), yes/no (% obese)	4/37 (9)	26/81 (24)	.049
Migraine, yes/no (% migraineurs)	7/36 (16)	19/88 (15)	.829
Needle 22GS/22GQ (% atraumatic needle)	13/30 (30)	61/47 (56)	.004
Number of LP attempts before succeeding	1.5 ± 0.7	1.4 ± 0.8	.623^a^
Opening pressure, cm H_2_O	15.8 ± 4.9	17.2 ± 4.6	.117
CSF removed, ml	7.7 ± 3.4	8.5 ± 3.8	.205^a^
CSF glucose, mmol/L	3.4 ± 0.3	3.5 ± 0.5	.193^a^
CSF total protein, mg	0.4 ± 0.1	0.4 ± 0.3	.288^a^
CSF WBCs per mm^2^	4.2 ± 6.8	5.8 ± 25.8	.148^a^

Abbreviations: 22GQ, 22‐gauge Quincke needle; 22GS, 22‐gauge Sprotte needle; BMI, body mass index; CSF WBCs, white blood cells in cerebrospinal fluid; CSF, cerebrospinal fluid; LP, lumbar puncture; PDPH, postdural puncture headache.

^a^ Nonparametric test (Mann–Whitney *U* test) used.

**TABLE 2:** IHS classification of PDPH (second edition)

Diagnostic criteria
Headache that worsens within 15 min after sitting or standing and improves within 15 min after lying, with at least one of the following and fulfilling criteria C and D: Neck stiffness Tinnitus Hypacusia Photophobia Nausea
Dural puncture has been performed
Headache develops within 5 days after dural puncture
Headache resolves either^a^: Spontaneously within 1 week Within 48 hr after effective treatment of the spinal fluid leak (usually by epidural blood patch)

Note

http://ihs‐classification.org/en/.

^a^ In 95% of cases, this is so. When headache persists, causation is in doubt.

### 2.7 Time frame

The study was conducted between February 2012 and March 2019.

### 2.8 Statistical analysis

The null hypothesis states that the incidence of PDPH after dLP with a 22G atraumatic needle is not different from the incidence of PDPH after dLP with a 22G traumatic needle. However, in previous studies, the incidence of PDPH was significantly higher in the cutting needle group than in the atraumatic group, from 36% versus 3% (Lavi et al., 2006) to 24.4% versus 12.2%. (Strupp et al., 2001) With a 90% power of achieving a significant result at the 5% level, the sample size to demonstrate an overall preference rate of 70% or more for diagnostics with 22G atraumatic needle, the calculated (Altman, 1991) sample size (for each sample separately) was 73. To allow for possible exclusions, loss to follow‐up, and possibly overestimating the superiority of the use of atraumatic needles, we decided that a total sample of 172 would be appropriate. Data were analyzed with IBM SPSS Statistics, version 26. Independent‐samples *t* test was used to analyze continuous variables. Some of the data were log‐transformed to fit the normal distribution; if not, the nonparametric Mann–Whitney *U* test was used. Categorical variables were compared using the chi‐squared test and presented as numbers and percentages. All tests were 2‐sided. Relative risk with its confidence interval was also calculated. Statistical significance was set at *p* < .05.

## 3 RESULTS

Due to somewhat slow recruitment, a few wrong inclusions, and loss of investigators in the initial phase, the study lasted longer than originally anticipated. The number of eligible patients assessed for participation was not recorded, nor the number of subjects not meeting the inclusion criteria. None of the patients asked for participation declined. In total, 172 patients were recruited and randomized. Of these, 21 patients were excluded after allocation; eleven due to wrong inclusion, seven because of procedural difficulties and needle switch, one dLP failed with no further attempts, one was lost to follow‐up, and one withdrew without reason (Figure 1). Demographics and baseline characteristics of the included are shown in Table 1 and that of the excluded are in Table 3.

**TABLE 3:** Demographic data excluded population

Excluded patients (*n* = 21^a^)	PDPH (*n* = 3)	No PDPH (*n* = 17)	p‐value
Mean age, years ± *SD*	34.7 ± 8.7	48.6 ± 15.1	0.143
Sex, males/females (% males)	1/2 (33)	12/5 (71)	0.201
Weight, kg	95 ± 42.4	80.1 ± 16.8	0.323^b^
Height, m	1.8 ± 0.08	1.8 ± 0.08	0.661^b^
Mean BMI, kg/m^2^	29 ± 10.6	25.9 ± 4.4	0.427^b^
BMI obese (BMI ≥ 30), yes/no (% obese)	1/2 (33)	1/16 (59)	0.31
Migraine, yes/no (% migraineurs)	0/3 (0)	4/13 (24)	0.559
Needle 22GS/22GQ (% atraumatic needle)	2/1 (67)	9/8 (53)	0.51
Number of LP attempts before succeeding	3.7 ± 2.5	2.8 ± 2.3	0.565^b^
Opening pressure, cm H_2_O	18.8 ± 2.5	16.4 ± 5.9	0.598^b^
CSF removed, ml	7.7 ± 2.5	6.7 ± 2.2	0.48
CSF glucose, mmol/L	3.4	3.5 ± 0.2	0.752^b^
CSF total protein, mg	0.4 ± 0.1	0.5 ± 0.2	0.284
CSF WBCs per mm^2^	4.3 ± 4.0	12.2 ± 40.7	0.748^b^

Abbreviations: 22GQ, 22‐gauge Quincke (traumatic needle); 22GS, 22‐gauge Sprotte (atraumatic needle); BMI, body mass index; CSF WBCs, white blood cells in the cerebrospinal fluid; CSF, cerebrospinal fluid; LP, lumbar puncture; PDPH, postdural puncture headache.

^a^ PDPH was not registered in one patient where LP failed and no CSF was obtained.

^b^ Nonparametric test (Mann–Whitney *U* test) used.

**FIGURE 2:**
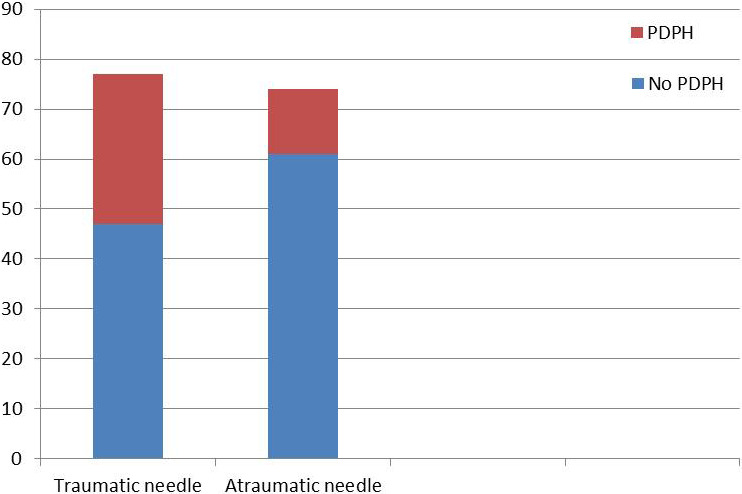
Bar plot showing the distribution of PDPH when using traumatic versus atraumatic needle

Of included patients, 77 were lumbar punctured with 22GQ and 74 with 22GS. In total, 43 patients developed a headache that met the IHS criteria for PDPH. Of the patients with PDPH, 13 were punctured with the atraumatic needle and 30 with the traumatic needle (Figure 2). The incidence of PDPH among the patients punctured with 22GS was thus 18%, and significantly lower than among the patients punctured with 22GQ, which was 39% (*p* = .004). The relative risk of PDPH for patients punctured with 22GS was 0.45 (95% CI 0.26–0.80). Absolute risk reduction was 21%. The patients who developed PDPH had a significantly lower BMI and weight (*p* = .012, *p* = .035) compared with the group who did not develop PDPH. We did not find any significant difference related to age (*p* = .064), sex (*p* = .239), height (*p* = .857), opening pressure (*p* = .117), operators (*p* = .148), amount CSF removed (*p* = .205), number of attempts (*p* = .623), or premorbid episodic migraine (*p* = .829). Two patients (4.7%) had to be treated with an EBP (both punctured with 22GQ).

Of the excluded patients, there were seven randomized to 22GS, who finally had to be punctured with 22GQ. Six of them were classified as being overweight, four with a body mass index (BMI) ≥ 30.

## 4 DISCUSSION/CONCLUSION

The incidence of PDPH reported in previous studies varies greatly and depends on a number of factors, including gender, age, BMI, premorbid headache, technical construct, and especially the gauge and the tip of the spinal needle (Alstadhaug et al., 2012). Despite moderate (Arevalo‐Rodriguez et al., 2017)‐to‐strong (Nath et al., 2018) evidence to support the use of atraumatic needles to reduce the incidence of PDPH, the practice does not seem to have been adopted in Europe. In a retrospective study of all LPs (*n* = 6,594) performed in two French university hospitals in 2014, only 8% were performed with the use of atraumatic needles. (Moisset et al., 2016) The use among British neurologists is probably not much higher (Davis et al., 2016). Our data confirm the result of previous studies; the use of 22GS spinal needles causes significantly fewer patients having PDPH.

As stated earlier, we believe that poor evaluation of the patientsˈ headaches is one of the greatest biases in previous RCTs. Furthermore, just a couple of studies published in the last 10 years were RCTs where patients were having dLP (Castrillo et al., 2015; Salzer et al., 2020), and not lumbar punctured because of other indications such as myelography or anesthesia. Most of the older RCTs have less than 100 participants (Lavi et al., 2006; Luostarinen et al., 2005; Thomas et al., 2000) except for the study by Strupp et al. (2001) There have also been studies published during the past decade where one has compared different needle sizes, for example, 22GQ with 25GS. (Engedal et al., 2015; Salzer et al., 2020) In these studies, it could be challenging to assess the effect of the atraumatic needle.

History of chronic headache poses a higher risk of PDPH. (Clark et al., 1996; Khlebtovsky et al., 2015) Chronic headaches were one of our exclusions criteria, which we believe is one of the strengths of our study. It is also known that the incidence of PDPH in patients older than 60 years is much lower than in the younger population and rare in children (Amorim et al., 2012; Evans et al., 2000; Khlebtovsky et al., 2015; Wadud et al., 2006). Patients included in our study were in the age‐group with the highest risk of developing PDPH. As also demonstrated in earlier studies (Evans et al., 2000; Kuntz et al., 1992; Lavi et al., 2006; Lynch et al., 1991; Wadud et al., 2006), we found that low BMI causes increased prevalence of PDPH. Our data seem to indicate that the female gender is a risk factor, however not significantly. Previous studies have shown that women are twice as much at risk of getting PDPH as men (Dripps & Vandam, 1954; Evans et al., 2000). Patients with premorbid episodic migraine were not at a higher risk of developing PDPH.

Our study was extended over a relatively long time period, and 12% of allocated participants were eventually excluded from the final outcome analyses. Including these, however, does not change the results. In the early phase of the study, recruitment and LPs were performed by physicians with variable clinical experiences. This caused wrong inclusion of a few patients. After 2014, however, exclusion of patients was only due to unsuccessful dLP and the need for needle switch. Seven patients randomized to 22GS had to switch to 22GQ, six of them classified as being overweight. The greater failure rate of 22GS in patients with a high BMI has been documented in other studies (Castrillo et al., 2015; Thomas et al., 2000). Lack of experience performing dLP on patients with high BMI can, however, be an explanation to this as the learning curve between traumatic and atraumatic LP is not different (Vakharia & Lote, 2012), and the fact that residents report preferring using atraumatic needle after completing training using a simulator (Tung, 2013).

It seems like the use of the traumatic needle is still frequent among neurologists, particularly in Europe (Davis et al., 2016; Moisset et al., 2016). The anesthesia community in the United States changed their practice to the use of atraumatic needles in the 1990s, despite the fact that many studies demonstrated the advantage of noncutting needles years before. The neurologic community in the United States was apparently not influenced by these studies, and they spent many years to change their practice. In 2002, atraumatic needles were introduced and made available at the Department of Neurology at the Mayo Clinic in Arizona. Over the years, the use of the atraumatic needles slowly increased among the American neurology community (Arendt et al., 2009). We hope that our study finally will cause other neurological communities to adopt this practice as well.

In conclusion, the use of 22G atraumatic needle for diagnostic lumbar puncture reduces the incidence of postdural puncture headache with almost 50% when compared with the use of 22G traumatic needle.

## CONFLICT OF INTEREST

The authors have nothing to disclose.

## AUTHOR CONTRIBUTION

Ane Skaare Sjulstad analyzed and interpreted the data, performed procedures and acquisition of data, and drafted the manuscript. Francis Odeh designed and conceptualized the study, performed procedures and acquisition of data, revised the manuscript for intellectual content, and approved the final version to be published. Farid K. Baloch played major role in performing procedures and acquisition of data, revised the manuscript for intellectual content, and approved the final version to be published. Diana H. Berg performed procedures and acquisition of data, revised the manuscript for intellectual content, and approved the final version to be published. Kathrine G. Arntzen performed procedures and acquisition of data, revised the manuscript for intellectual content, and approved the final version to be published. Karl B. Alstadhaug designed and conceptualized the study, analyzed and interpreted data, revised the manuscript for intellectual content, and approved the final version to be published.

## AUTHOR GUARANTEE STATEMENT

All coauthors have made a substantial contribution to the design, data collection, analysis of the research, and the drafting of the manuscript and have reviewed and accepted the contents of the manuscript prior to its submission.

### Peer Review

The peer review history for this article is available at https://publons.com/publon/10.1002/brb3.1886.

## Data Availability

Data will be provided to other investigators upon request made to the corresponding author if ethical approval is granted.
